# Antibiotic-related gut dysbiosis induces lung immunodepression and worsens lung infection in mice

**DOI:** 10.1186/s13054-020-03320-8

**Published:** 2020-10-15

**Authors:** Rodrigue Dessein, Marvin Bauduin, Teddy Grandjean, Rémi Le Guern, Martin Figeac, Delphine Beury, Karine Faure, Christelle Faveeuw, Benoit Guery, Philippe Gosset, Eric Kipnis

**Affiliations:** 1 Univ. Lille, CNRS, Inserm, CHU Lille, Institut Pasteur de Lille, U1019-UMR9017-CIIL-Centre d’Infection et d’Immunité de Lille, Lille, France, University Lille, F-59000 Lille, France; 2 CHU Lille, Institut Pasteur de Lille, Inserm, CNRS, UMR2014—US41—PLBS-6 Plateformes Lilloises de Biologie & Santé, University Lille, F-59000 Lille, France; 3 Infectious Diseases Service, Department of Medicine, University Hospital and University of Lausanne, Lausanne, Switzerland

**Keywords:** Antibiotics, Dysbiosis, *Pseudomonas aeruginosa*, Murine model, Flt3-ligand, Fecal microbial transplant

## Abstract

**Background:**

Gut dysbiosis due to the adverse effects of antibiotics affects outcomes of lung infection. Previous murine models relied on significant depletion of both gut and lung microbiota, rendering the analysis of immune gut-lung cross-talk difficult.

Here, we study the effects of antibiotic-induced gut dysbiosis without lung dysbiosis on lung immunity and the consequences on acute *P*. *aeruginosa* lung infection.

**Methods:**

C57BL6 mice received 7 days oral vancomycin-colistin, followed by normal regimen or fecal microbial transplant or Fms-related tyrosine kinase 3 ligand (Flt3-Ligand) over 2 days, and then intra-nasal *P*. *aeruginosa* strain PAO1. Gut and lung microbiota were studied by next-generation sequencing, and lung infection outcomes were studied at 24 h. Effects of vancomycin-colistin on underlying immunity and bone marrow progenitors were studied in uninfected mice by flow cytometry in the lung, spleen, and bone marrow.

**Results:**

Vancomycin-colistin administration induces widespread cellular immunosuppression in both the lung and spleen, decreases circulating hematopoietic cytokine Flt3-Ligand, and depresses dendritic cell bone marrow progenitors leading to worsening of *P*. *aeruginosa* lung infection outcomes (bacterial loads, lung injury, and survival). Reversal of these effects by fecal microbial transplant shows that these alterations are related to gut dysbiosis. Recombinant Flt3-Ligand reverses the effects of antibiotics on subsequent lung infection.

**Conclusions:**

These results show that gut dysbiosis strongly impairs monocyte/dendritic progenitors and lung immunity, worsening outcomes of *P*. *aeruginosa* lung infection. Treatment with a fecal microbial transplant or immune stimulation by Flt3-Ligand both restore lung cellular responses to and outcomes of *P*. *aeruginosa* following antibiotic-induced gut dysbiosis.

**Supplementary information:**

**Supplementary information** accompanies this paper at 10.1186/s13054-020-03320-8.

## Background

Recent advances in high-throughput bacterial sequencing, allowing the large-scale study of the microbial communities in humans and experimental models, led to the study of the bacteria composing this flora, since termed “microbiome” (or microbiota), as well as their pathologic alterations, termed dysbiosis (or dysbioses) [1]. Dysbiosis has been shown to be responsible for altered immune responses against pathogens [2]. The disruption of this microbiome-driven immune homeostasis by antibiotics is considered a mechanism for the deleterious effects of antibiotics beyond the gut, such as predisposition to allergic lung diseases [3]. Non-culture-based methods have shown that the human microbiome includes bacterial communities in body compartments other than the gut, such as the lung. Therefore, the microbiome-driven immune cross-talk between the gut and the lung, or “gut-lung axis,” involves both gut and lung microbiota [4]. However, these studies are based on models of antibiotic-induced dysbiosis that rely on very broad-spectrum antibiotics using associations of 4 to 5 antibiotics, many with systemic diffusion. Not only are such models far from mirroring clinical situations, but they also result in the form of dysbiosis with significant overall depletion [5], and deplete both the gut and lung microbiota [5, 6]. Therefore, it is difficult to decipher the respective roles of the lung and gut microbiota on lung immunity and its impact on lung infection.

We aim to determine the effects of antibiotic-induced gut dysbiosis, without lung dysbiosis, on outcomes in a non-lethal murine model of acute *P*. *aeruginosa* lung infection and the underlying immune alterations in order to manage side effects of antibiotics.

## Methods

### Experimental model and study design

**Fig. 1:**
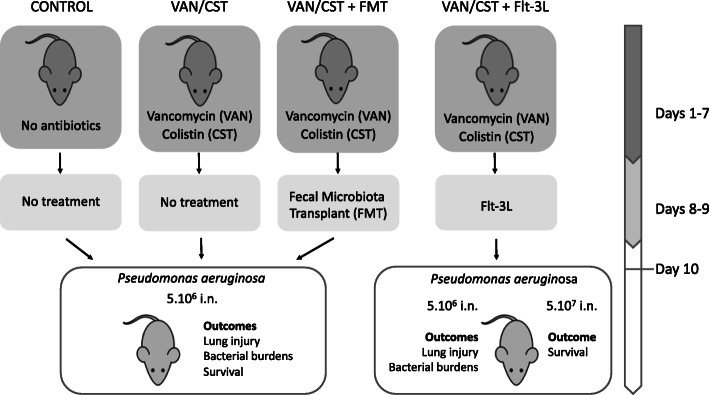
Experimental design and study groups

Gut dysbiosis was induced by treating 6–8-week-old C57BL/6 mice with non-absorbable antibiotics (vancomycin 0.5 g/L and colistin 0.15 g/L) administered in drinking water ad libitum for 7 days; the control animals received untreated drinking water. In groups treated with either immunomodulation (Flt3-Ligand) or microbiota modulation (fecal microbiota transplant), these interventions were performed on days 8 and 9 as described below. At day 10, mice were anesthetized briefly with inhaled sevoflurane, and acute sublethal pneumonia was performed by intra-nasal instillation of PAO1 at 5.10^6^ CFU (or lethal pneumonia with PAO1 at 5.10^7^ CFU in a subset of experiments). Control mice were intra-nasally instilled with phosphate buffer saline (PBS) instead of bacteria. For all experiments except survival studies, mice were euthanized at 24 h after PAO1 inoculation. Survival studies followed mice over 96 h, upon which surviving animals, if any, were euthanized. The experimental design and study groups are illustrated in Fig. 1.

All animal experiments were performed according to national and local institutional committee for the use of experimental animals’ guidelines and approval from the national experimental ethics committee (APAFIS number 7166).

Details concerning bacteria strain and culture, inoculums, and animal provenance and husbandry can be found in supplementary materials.

### Study outcomes: lung injury, bacterial burdens, and survival

In the abovementioned experimental study groups (Fig. 1), the following outcomes were studied. Alveolar-capillary barrier permeability was evaluated by measuring fluorescein isothiocyanate (FITC)-labeled-albumin leakage from the vascular compartment to the alveolar-interstitial compartment, expressed as permeability index, as previously described [7]. Bronchoalveolar lavage (BAL) was performed through washing with a total of 1.5 mL PBS. Lung and spleen bacterial burdens were studied by culturing sequential dilutions of homogenates on agar plates. In survival experiments, mice were followed up for up to 96 h. Experimental details are provided in supplementary materials.

### Underlying mechanisms: gut and lung microbiota, lung and spleen cellular responses, hematopoietic factors/cytokines, and bone marrow progenitors

Lung and gut microbiota were studied by next-generation sequencing through bacterial ribosomal 16S gene sequencing on an Illumina Miseq (Illumina, San Diego, CA). Detailed processes and bioinformatic analyses are provided in the supplemental materials.

Lung and spleen cellular responses and bone marrow progenitors were studied through flow cytometry on organ homogenates on a LSR Fortessa (BD Biosciences, San Jose CA) using appropriate cell-type antibody panels. The gating strategy for neutrophils, antigen-presenting cells, and the significant populations of lymphocytes in the lung are presented in the supplemental figure 1.

Bone marrow progenitor cell populations were analyzed within the CD45+ phenotype with a BD LSR Fortessa (BD Biosciences) using antibody panels gating strategies and bioinformatic analyses according to the targeted cell surface phenotypes (fully detailed in supplementary materials).

### Modulation: fecal microbiota transplant and Flt-3L administration

For fecal microbiota transplantation (FMT), fecal pellets from untreated mice were suspended in PBS (1 fecal pellet/1 mL of PBS). A total of 200 μL of the resuspended pooled fecal material were administered by oral gavage.

Flt3-Ligand treatment (Ozyme, Saint-Cyr L’Ecole, France) was given by intraperitoneal injection over 2 consecutive days after antibiotic treatment was stopped, for a total of 10 μg/mice.

### Statistical analyses

Statistical analysis was carried out using Prism 5 software (Graph-Pad Software, San Diego, CA). Values are expressed as mean ± SD. Comparison between groups was analyzed with nonparametric ANOVA followed by Dunn’s multiple comparisons post hoc tests. Survival curves were analyzed using the Log-rank (Mantel-Cox) test. Significance was accepted at *p* < 0.05.

## Results

### Prior oral non-absorbable antibiotic-induced gut dysbiosis worsens outcomes of *P*. *aeruginosa* lung infection

**Fig. 2:**
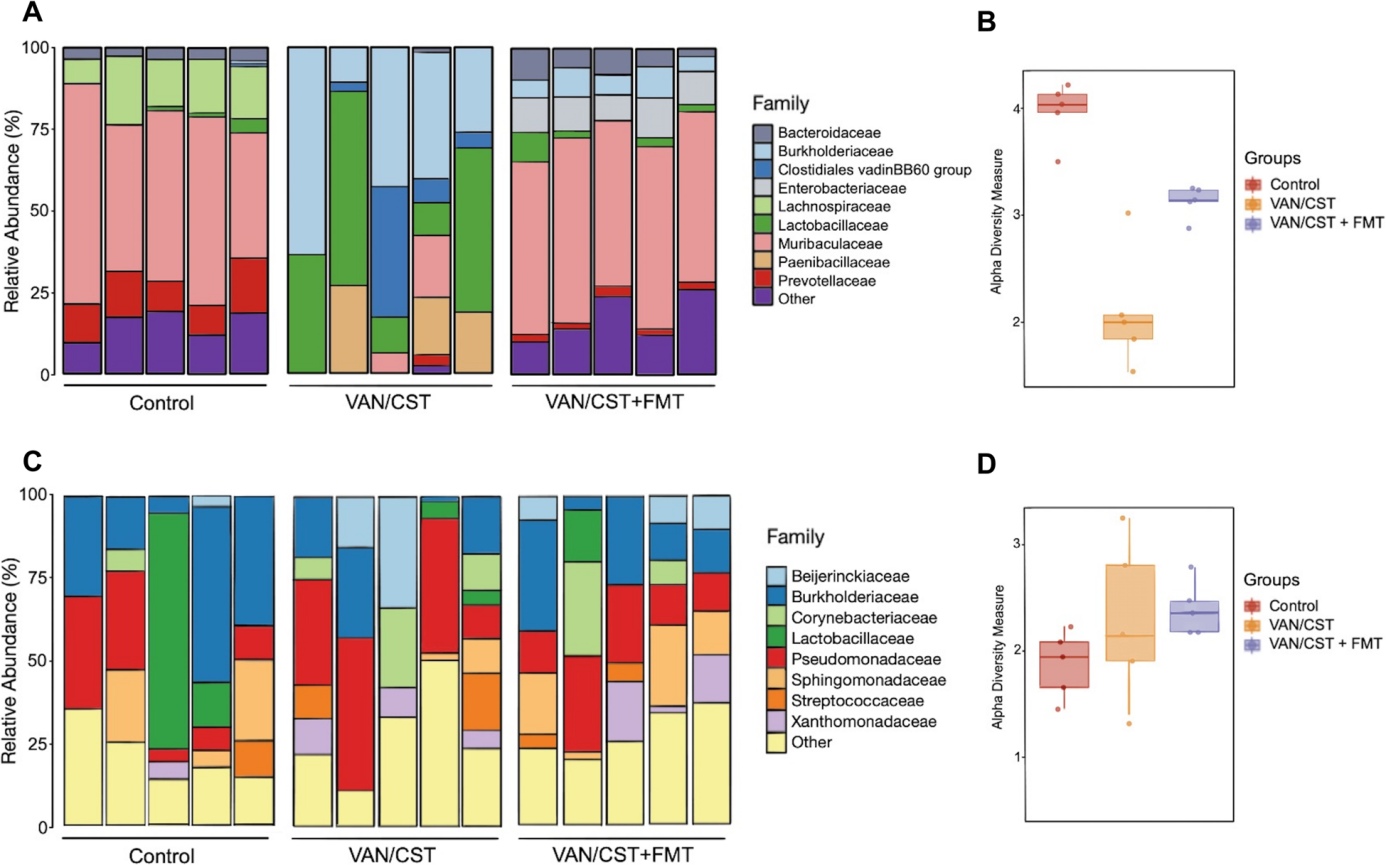
Gut microbiota and lung infection by *P*. *aeruginosa*. **a** Bacterial family as percentages of sequenced 16S rDNA in the stool of uninfected mice treated or not by oral vancomycin (VAN) and colistin (CST) or fecal microbiota transplant (FMT). **b** Alpha diversity (Shannon index) of distinguishable taxa in sequenced stool samples. **c** Bacterial family as percentages of sequenced 16S rDNA in the lung of uninfected mice treated or not by oral vancomycin (VAN) and colistin (CST) or fecal microbiota transplant (FMT). **d** Alpha diversity (Shannon index) of distinguishable taxa in sequenced stool samples. For all microbiota experiments, there are 5 mice per group

First, we assessed if oral non-absorbable antibiotics induced gut dysbiosis. The gut microbiota from antibiotic-treated mice before *P*. *aeruginosa* intra-nasal challenge was altered compared to control (Fig. 2a). We also observed an eradication of several bacterial families like *Muribaculaceae*, *Prevotellaceae*, and *Lachnospiraceae* and an expansion of *Burkholderiaceae*, *Clostridiales*, and *Lactobacillaceae*. Eradication of several bacterial families was associated with a decrease of gut microbiota diversity (Fig. 2b). These modifications were observed with a decrease of only one log of 16srDNA compared to untreated mice (supplemental figure 2). As expected, we did not observe any modification of the effect of prior oral non-absorbable on the composition and the diversity of the lung microbiota before *P*. *aeruginosa* infection (Fig. 2c, d).

**Fig. 3:**
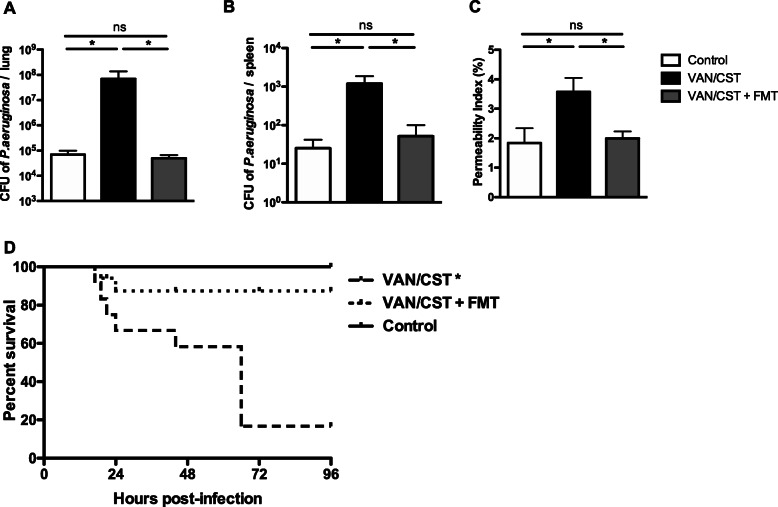
Outcomes of lung infection by *P*. *aeruginosa*. **a**, **b** Lung and spleen bacterial loads and **c** alveolar-capillary barrier permeability measured 24-h after intra-nasal sublethal *P*. *aeruginosa* (PAO1), in mice previously treated or not (controls) with 7-day oral vancomycin/colistin (VAN/CST) followed by fecal microbiota transplant (VAN/CST + FMT) or not. **d** 72-h survival of mice challenged with sublethal *P*. *aeruginosa* (PAO1) treated or not (controls) with 7-day oral vancomycin/colistin (VAN/CST) followed by fecal microbiota transplant (VAN/CST + FMT) or not. For all experiments, there are 5 mice per group, except for mortality, and 10 mice per group in duplicate; results are shown as mean ± SD; *p < 0.05

Then, we assessed if prior oral non-absorbable antibiotic modified outcomes of *P*. *aeruginosa* acute pneumonia. Antibiotics administered in drinking water 7 days before *P*. *aeruginosa* intra-nasal challenge were associated with worse infection outcomes (Fig. 3). *P*. *aeruginosa* load increased^s^ by 10^3^ CFU and 10^2^ in the lung and in the spleen, respectively, in antibiotic condition (Fig. 3a, b). Lung injury was increased in antibiotic pre-treated mice when compared to untreated controls (3.5 ± 1.4% vs. 1.8 ± 1.1%, *p* < 0.05) (Fig. 3c). Finally, the survival of antibiotic-treated mice survival decreased to 20% from 100% in untreated controls (*p* < 0.05) (Fig. 3d). Overall, acute sublethal *P*. *aeruginosa* pneumonia became lethal in antibiotic-treated mice compared to untreated controls.

To demonstrate the causality of gut dysbiosis in worse outcomes of *P*. *aeruginosa*-infected mice, we treated dysbiosis using fecal microbiota transplant (FMT). Following dysbiosis-inducing antibiotics, FMT also restored the composition and the diversity of the gut microbiota equivalent to controls (Fig. 2a, b) and did not modify the composition and the diversity of the lung microbiota (Fig. 2c, d). Of note, *P*. *aeruginosa* intra-nasal challenge did not further modify the gut microbiota shift observed in antibiotic-treated mice (supplemental figure 3). As expected, *P*. *aeruginosa* intra-nasal challenge resulted in a lung microbiota dominated by *Pseudomonadaceae* (supplemental figure 3). Finally, FMT before *P*. *aeruginosa* inoculation restored pneumonia outcomes to those of control mice (Fig. 3a–d).

### Oral non-absorbable antibiotic-induced gut dysbiosis results in widespread lung and spleen immune depression associated with altered myelopoiesis

Next, we assessed whether antibiotic-induced gut dysbiosis had any effects on baseline lung and circulating cellular immune profiles as a potential mechanism underlying worse outcomes following *P*. *aeruginosa* lung infection.

**Fig. 4:**
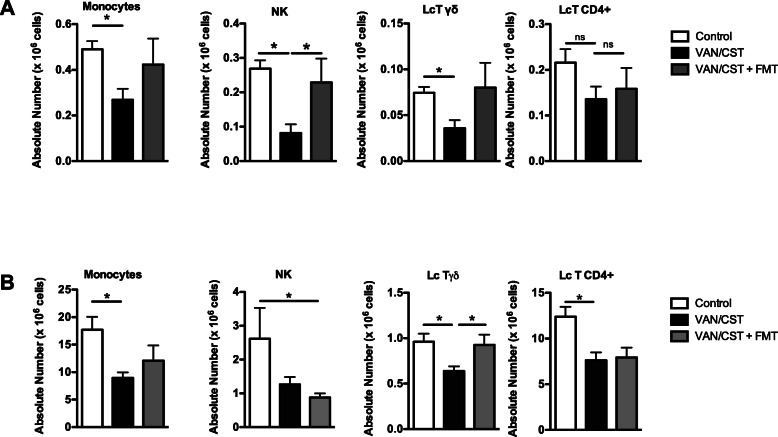
Effects of oral non-absorbable antibiotics on lung and spleen immune cell populations. **a** Flow cytometry of immune cells from lung homogenates in mice treated or not (controls) with 7-day oral vancomycin/colistin (VAN/CST) followed by fecal microbiota transplant (VAN/CST + FMT) or not. **b** Flow cytometry of immune cells from spleen homogenates in mice treated or not (controls) with 7-day oral vancomycin/colistin (VAN/CST) followed by fecal microbiota transplant (VAN/CST + FMT) or not. For all experiments, group size is 5 mice per group; results are shown as mean ± SD; *p < 0.05

Cell population analysis in lung tissue showed that antibiotics resulted in widespread depression of lung cellular immunity with a significant decrease in macrophages, cDC2, inflammatory monocytes, neutrophils, γδ-T cells, NKs, and iNKT cells (Fig. 4a). FMT restored most of these alterations in macrophages, cDC2, inflammatory monocytes, neutrophils, and iNKT cells (Fig. 4a), establishing a significant role of antibiotic-induced gut dysbiosis in this widespread depression of the lung immune response. Likewise, antibiotics resulted in widespread depression of most studied spleen immune cell populations (*p* < 0.05 except for a trend in NK cells), also partially restored by FMT (Fig. 4b).

**Fig. 5:**
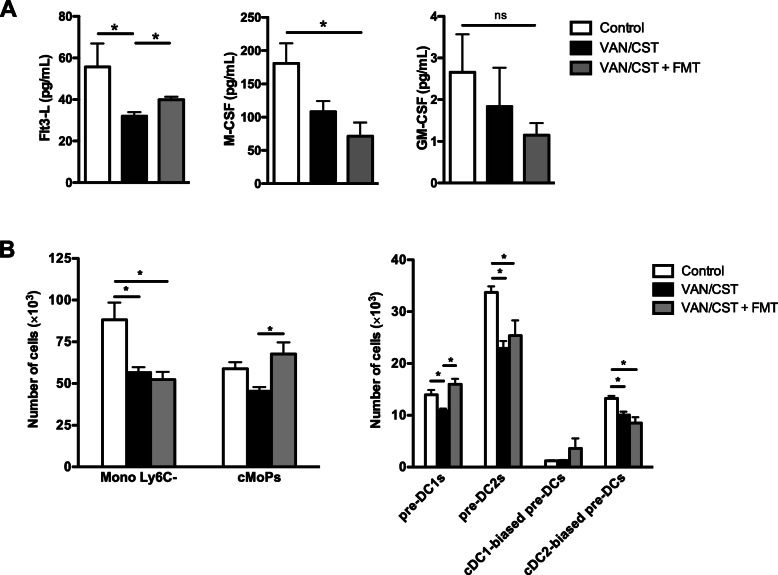
Effects of oral non-absorbable antibiotics on hematopoietic factors and bone marrow monocyte/dendritic cell progenitors. **a** ELISA of hematopoietic factors in serum of mice treated or not (controls) with 7-day oral vancomycin/colistin (VAN/CST) followed by fecal microbiota transplant (VAN/CST + FMT) or not. **b** Flow cytometry of monocyte/DC progenitors in the bone marrow in mice treated or not (controls) with 7-day oral vancomycin/colistin (VAN/CST) followed by fecal microbiota transplant (VAN/CST + FMT) or not. For all experiments, group size is 5 mice per group; results are shown as mean ± SD; *p < 0.05

Because the spleen is a hematopoietic organ in mice, we sought to determine the effects of antibiotics on immune cell hematopoietic factors, specifically on circulating serum levels of GM-CSF, M-CSF, and Flt3-Ligand (Fig. 5a). While circulating levels of GM-CSF were at the detection threshold (1 pg/mL) and remained unaltered, antibiotics induced a significant decrease in Flt3-Ligand, and only a trend in M-CSF decrease. Given the known role of Flt3-Ligand as a major hematopoietic stimulating factor, mainly for monocyte and DC progenitors, we studied the effects of antibiotics and FMT on bone marrow monocyte and DC progenitors (Fig. 5b and supplemental figure 5). We found that antibiotics were associated with a significant decrease in bone marrow progenitors specific to resident monocytes (MonoLy6C-) and several specific to DCs (total pre-DCs, pre-DCs 1 and 2, and cCD2-biased pre-DCs). Among these, FMT following antibiotics restored levels of pre-DCs 1. FMT also stimulated the expansion of bone marrow progenitors common to monocytes/macrophages/DCs (MDP), common dendritic cell progenitors (CDP), and specific to monocytes and macrophages (cMoPs). Our results suggest that the effects of antibiotic-induced dysbiosis on monocyte/macrophage and DC progenitors may be involved in the widespread antibiotic-induced lung immune depression associated with worse *P*. *aeruginosa* lung infection outcomes.

### Hematopoietic cytokine Flt3-L stimulates bone marrow progenitor and lung immune cell expansion and restores outcomes of *P*. *aeruginosa* lung infection following oral non-absorbable antibiotics

**Fig. 6:**
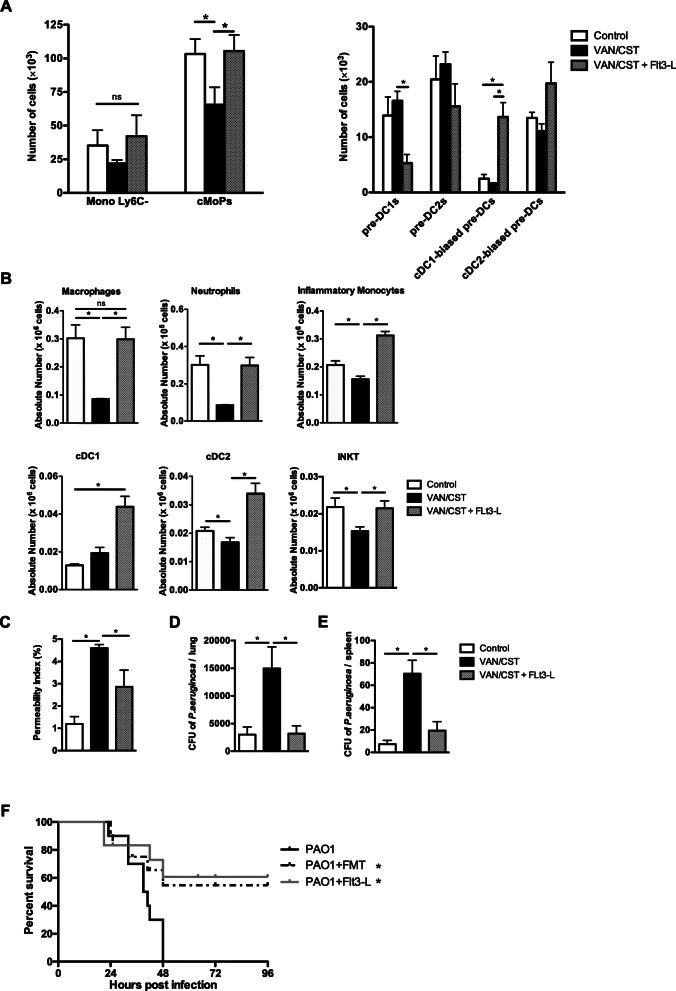
Effects of Flt3-Ligand on lung immune cell populations and on outcomes of lung infection. **a** Flow cytometry of monocyte/DC progenitors in the bone marrow of mice treated or not (controls) with 7-day oral vancomycin/colistin (VAN/CST) followed by systemic Flt3-Ligand administration (VAN/CST + Flt3-L) or not. **b** Flow cytometry of immune cells from lungs of mice with 7-day oral vancomycin/colistin (VAN/CST) followed by systemic Flt3-Ligand administration (VAN/CST + Flt3-L) or not. **c**, **d** Lung and spleen bacterial counts and **e** alveolar-capillary barrier permeability, measured in mice treated or not (controls) with 7-day oral vancomycin/colistin (VAN/CST) followed by systemic Flt3-Ligand administration (VAN/CST + Flt3-L) or not. For all experiments, group size is 5 mice per group; results are shown as mean ± SD; *p < 0.05. **e** Survival of mice after lethal *P*. *aeruginosa* pneumonia treated or not by FMT or Flt3-L; group size is 10 mice per group; *p < 0.05

Systemic Flt3-L following antibiotics stimulated the expansion of several progenitors (MDPs, CDPs, and cDC-1-biased pre-DCs) (Fig. 6a and supplemental figure 6). Flt3-Ligand administration partially restored or overstimulated the expansion of depressed alveolar macrophages, cDC2, inflammatory monocytes, neutrophils, NK, and iNKT (Fig. 6b and supplemental figure 6).

Finally, the effects of Flt3-Ligand (Flt3-L) administration following antibiotics on the outcomes of sublethal *P*. *aeruginosa* lung infection were similar to those of FMT. Thus, Flt3-L administration led to a decrease permeability index in VAN/CST mice group compared to VAN/CST + FLT-3 mice group (Fig. 6c). Additionally, Flt3-L administration led to a decrease of *P*. *aeruginosa* load in both lung and spleen in VAN/CST mice group compared to VAN/CST + Flt-3L mice group to a level similar to the controls (Fig. 6d, e). Finally, Flt3-Ligand was also associated with decreased mortality in a lethal *P*. *aeruginosa* lung infection model, from 100 to 40% (*p* < 0.05) similar to the effects of FMT (Fig. 6f).

## Discussion

Our results show that oral non-absorbable antibiotics induce gut dysbiosis, without lung dysbiosis, leading to a widely depressed lung immune cellular response responsible for worse outcomes of subsequent *P*. *aeruginosa* lung infection. These effects involve bone marrow progenitor depression and are susceptible to therapeutic immunomodulation by the hematopoietic cytokine Flt3-L as well as fecal microbial transplant.

Our study shares limitations inherent to the experimental model. Ours is a murine model that does not strictly mimic a clinical situation and cannot therefore be extrapolated to clinical practice. However, animal models allow studying complex interactions such as the gut-bone marrow-lung axis without the added complexities related to the heterogeneity of patients. Furthermore, our choice of oral non-absorbable antibiotics (vancomycin and colistin) while not representative of antimicrobial therapies administered to intensive care unit (ICU) patients, allowed us to differentiate the effects of antibiotics on the gut from the lung. Additionally, oral vancomycin and colistin are components of selective digestive decontamination (SDD), which is a relevant ICU situation. Technical limitations to microbiota analysis were a limited number of animals that was mitigated by very clear shifts and/or inversions observed. Last, animals were not randomized to study groups, while this may be a potential study bias; studied mice are a standard breeding lineage from large batches bred by an international supplier where breeding schemes help minimize the risk of systematic differences in baseline characteristics.

The first description linking altered gut microbiota and outcome of bacterial lung infection used germ-free mice infected with *Klebsiella pneumoniae* [8]. Several authors described afterward that the gut microbiota protects against respiratory infection by *Streptococcus pneumoniae*, *K*. *pneumoniae*, and *P*. *aeruginosa* [5, 6, 9]. However, these studies used very broad-spectrum antibiotic regimens, which lead to a significant depletion of overall bacterial 16srDNA in the gut. We observed a reduction from 10^6^ to 10^2^ gene copies/ng DNA [5], which is closer to “germ-free-like” models than to clinically relevant antibiotic regimens. In comparison, our model’s gut dysbiosis was mainly due to shifts in phyla, not to extreme overall depletion. We observed depletion of only 1 log copies 16 s rDNA (supplemental figure 3) rather than 4 log copies 16 s rDNA [5]. Therefore, our data suggest that eradicating some phyla and/or expansion of others is sufficient to alter the responses to lung infection independently of significant/complete overall gut microbiome depletion.

Conversely to lung dysbiosis associated with altered outcomes like asthma [10], our treatment did not significantly modify the lung microbiota, suggesting other links between the observed gut dysbiosis and lung outcomes. Since we used non-absorbable oral antibiotics, this treatment had no direct effect on the lung microbiota. In contrast, this may have been the case in other dysbioses induced by systemically diffusing antibiotics, as in the study of Robak et al. in which lung microbiota depletion was confirmed [5]. Therefore, lung dysbiosis is not a requirement for the deleterious effects of prior antibiotics, and lung dysbiosis may rather be another collateral damage when antibiotics diffuse systemically to the lung.

In previous studies in which antibiotic-induced gut dysbiosis was shown to worsen bacterial pneumonia outcomes, the study of lung immune responses was focused in scope. Indeed, assessment of lung immunity restricted to macrophages showed that macrophage function was impaired consecutively to antibiotic-induced gut dysbiosis and involved in worse outcomes in an *S*. *pneumoniae* pneumonia model [9]. Likewise, lung immunity assessment restricted to IgA-producing cells found that IgA production was impaired in the lung and involved in worse outcomes in a *P*. *aeruginosa* pneumonia model [5].

We conducted a broader assessment of the immune response after antibiotic-induced gut dysbiosis and observed widespread lung cellular immune depression. Immune depression included cells crucial to the clearance of *P*. *aeruginosa* from the lung, such as alveolar macrophages, non-conventional lymphocytes, and neutrophils. Bone marrow progenitors of monocytes and DC were depressed and partially corrected by FMT, suggesting the involvement of immune cell hematopoiesis. A link between antibiotic-induced dysbiosis and depressed hematopoiesis has been demonstrated. In a non-infectious model [11] and in mice infected by *Flaviviridae* [12].

We found that FMS-like tyrosine kinase 3 ligand (Flt3-Ligand) was significantly decreased by antibiotic-induced gut dysbiosis and partially restored by FMT. Flt3-Ligand is a cytokine that acts as a hematopoietic growth factor for early progenitors through its receptor, Flt3, in synergy with other cytokines [13]. When administered after antibiotics, Flt3-L (a) restored or stimulated the expansion of several monocytes and dendritic cells (DC) bone marrow progenitors, (b) restored or stimulated immune lung cell populations, (c) restored outcomes of sublethal *P*. *aeruginosa* lung infection, and (d) increased survival of lethal *P*. *aeruginosa* lung infection. Flt3-Ligand promotes the generation of a primarily myeloid cell containing colonies. Flt3-Ligand is essential to the generation of DC. Furthermore, Flt3-Ligand transgenic mice show a significant expansion of Flt3-positive cells and progenitors (myeloid cells, DCs, MPP, CMP, granulocyte-macrophage progenitors, CLP, and EPLM progenitors) [14]. Administration of human Flt3-Ligand into mice increases DC-marker positive cells in the bone marrow, liver, Peyer’s patches, thymus, peritoneum, and lung [15]. In a murine model of influenza A virus infection, Beshara et al. showed that Flt3-L overexpression reduced the dissemination of *S*. *pneumoniae* instilled into the lungs by enhancing bone marrow cDC progenitors and restoring lung cDCs [16]. These results suggest that its protective effect is not limited to *P*. *aeruginosa*.

Interestingly, recombinant human Flt3-L (CDX-301, Celldex Therapeutics, Hampton, NJ, USA) is currently undergoing over 30 clinical trials to treat various malignancies. Furthermore, given the FMT recent safety issues [17], it is of particular interest that deleterious effects of prior antibiotics on lung immunity may be modulated beyond the gut microbiome.

## Conclusion

Contrary to emerging controversial postulates [18], the gut microbiota, independently from the lung microbiota, might be crucial to developing an appropriate lung immune response as suggested by our results restricted to oral vancomycin-colistin and *P*. *aeruginosa* strain PAO1 in mice. In this specific murine model, treating antibiotic-induced dysbiosis through FMT or treating the associated lung immune depression through Flt3-Ligand restores *P*. *aeruginosa* (PAO1 strain) lung infection outcomes in mice and may be potential directions for translational research.

## Supplementary information

**Additional file 1 : Supplemental figure 1.** Gating strategy for neutrophils, antigen-presenting cells, and the significant populations of lymphocytes in the lung. The percentages of natural killer (NK), invariant NKT cells (iNKT), and T Lc with a TCRgd were determined. Among APC, we identified alveolar macrophages (AM), inflammatory monocytes, patrolling monocytes, interstitial macrophages (IM), and conventional dendritic cells (cDC)1 and 2.

**Additional file 2 : Supplemental figure 2**. Total bacterial 16SrDNA in the stool. Mice (*n* = 5) were treated by oral vancomycin (VAN) and colistin (CST) or not. Total 16srDNA expressed in relative quantity (fold-decrease) between VAN/CST and untreated controls (reference = 1).

**Additional file 3 : Supplemental figure 3**. Bacterial family as percentages of sequenced 16S rDNA in the lung and gut. Mice were treated or not by oral vancomycin (VAN), and colistin (CST) followed or not by fecal microbiota transplant (FMT) and infected or not by *P*. *aeruginosa* PAO1 (*n* = 2 per group).

**Additional file 4 : Supplemental figure 4**. Effects of oral non-absorbable antibiotics on lung and spleen immune cell populations. (A) Flow cytometry of immune cells from lung homogenates in mice treated or not (controls) with 7-days oral vancomycin/colistin (VAN/CST) followed by fecal microbiota transplant (VAN/CST + FMT) or not. (B) Flow cytometry of immune cells from spleen homogenates in mice treated or not (controls) with 7-days oral vancomycin/colistin (VAN/CST) followed by fecal microbiota transplant (VAN/CST + FMT) or not. All experiments, group size 5 mice per group, results are shown as mean ± SD; *: *p* < 0.05.

**Additional file 5 : Supplemental figure 5**. Effects of oral non-absorbable antibiotics on hematopoietic factors and bone marrow monocyte/dendritic cell progenitors. (A) ELISA of hematopoietic factors in serum of mice treated or not (controls) with 7-days oral vancomycin/colistin (VAN/CST) followed by fecal microbiota transplant (VAN/CST + FMT) or not, (B) Flow cytometry of monocyte/DC progenitors in the bone marrow in mice treated or not (controls) with 7-days oral vancomycin/colistin (VAN/CST) followed by fecal microbiota transplant (VAN/CST + FMT) or not. For all experiments, group size 5 mice per group; results are shown as mean ± SD; *: *p* < 0.05.

**Additional file 6 : Supplemental figure 6**. Effects of Flt3-Ligand on lung immune cell populations and on outcomes of lung infection. (A) Flow cytometry of monocyte/DC progenitors in the bone marrow of mice treated or not (controls) with 7-days oral vancomycin/colistin (VAN/CST) followed by systemic Flt3-Ligand administration (VAN/CST + Flt3-L) or not, (B) Flow cytometry of immune cells from lungs of mice with 7-days oral vancomycin/colistin (VAN/CST) followed by systemic Flt3-Ligand administration (VAN/CST + Flt3-L) or not. For all experiments, group size 5 mice per group; results are shown as mean ± SD; *: p < 0.05.

**Additional file 7.**

## Data Availability

Not applicable.
