# Supplementary material for: Antibiotic-related gut dysbiosis induces lung immunodepression and worsens lung infection in mice
Source: Crit Care. 2020 Oct 15;24:611. doi: 10.1186/s13054-020-03320-8 (PMC7574210; doi:10.1186/s13054-020-03320-8)
Supplement: Supplementary file 1 — Additional file 1 : Supplemental figure 1. Gating strategy for neutrophils, antigen-presenting cells, and the significant populations of lymphocytes in the lung. The percentages of natural killer (NK), invariant NKT cells (iNKT), and T Lc with a TCRgd were determined. Among APC, we identified alveolar macrophages (AM), inflammatory monocytes, patrolling monocytes, interstitial macrophages (IM), and conventional dendritic cells (cDC)1 and 2. [file 13054_2020_3320_MOESM1_ESM.pdf]

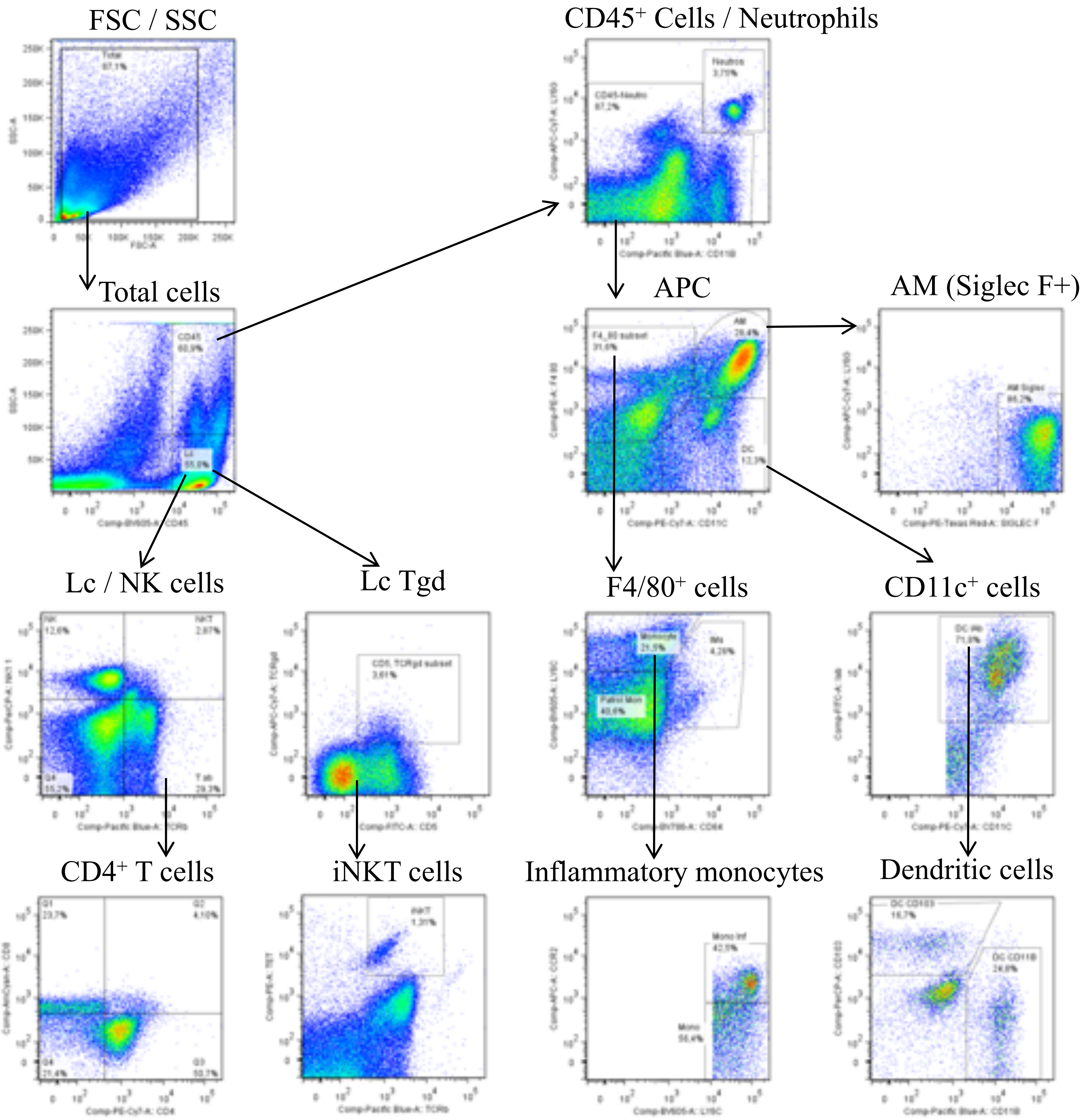
FSC / SSC
Total cells
CD45+ Cells / Neutrophils
APC
AM (Siglec F+)
Lc / NK cells
Lc Tgd
F4/80+ cells
CD11c+ cells
CD4+ T cells
iNKT cells
Inflammatory monocytes
Dendritic cells
