# Supplementary figures and images for: Antibiotic-related gut dysbiosis induces lung immunodepression and worsens lung infection in mice
Source: Crit Care. 2020 Oct 15;24:611. doi: 10.1186/s13054-020-03320-8 (PMC7574210; doi:10.1186/s13054-020-03320-8)

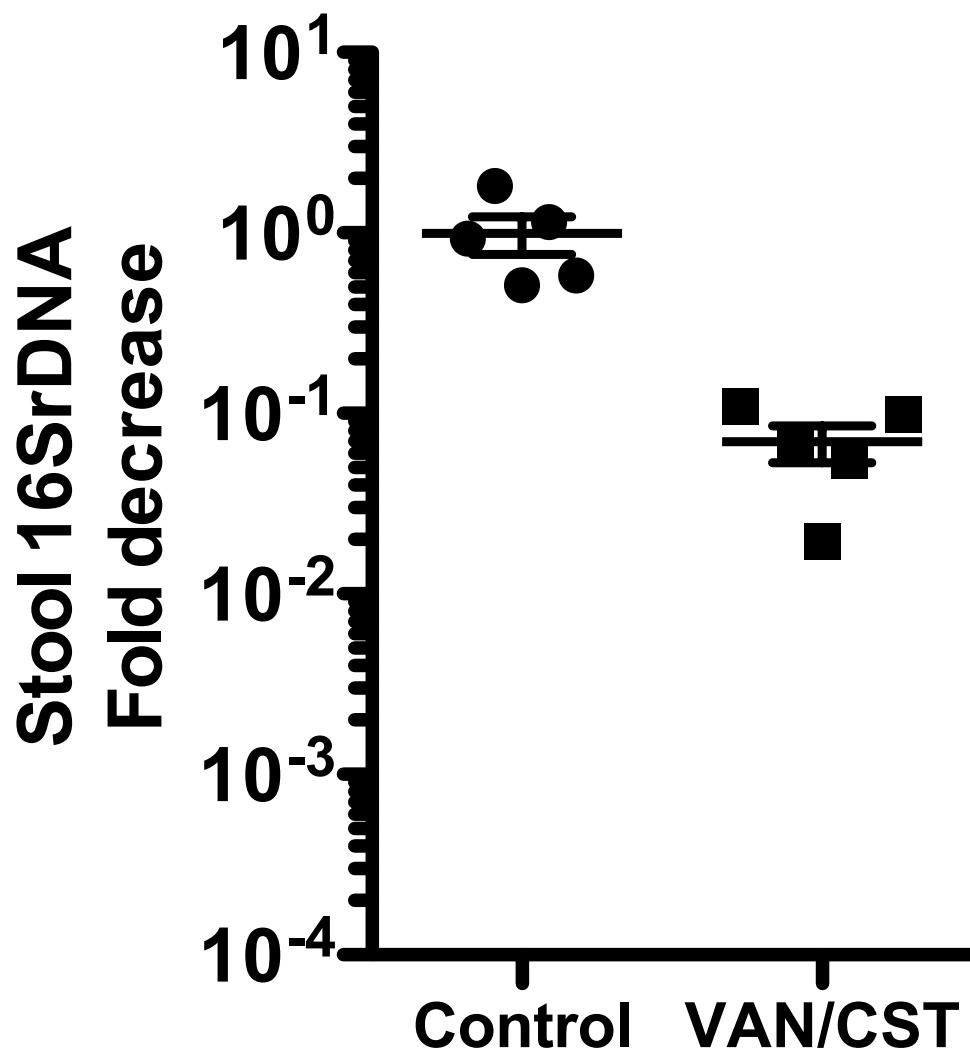

Supplement: Supplementary file 2 — Additional file 2 : Supplemental figure 2. Total bacterial 16SrDNA in the stool. Mice (n = 5) were treated by oral vancomycin (VAN) and colistin (CST) or not. Total 16srDNA expressed in relative quantity (fold-decrease) between VAN/CST and untreated controls (reference = 1). [file 13054_2020_3320_MOESM2_ESM.pdf]

**A**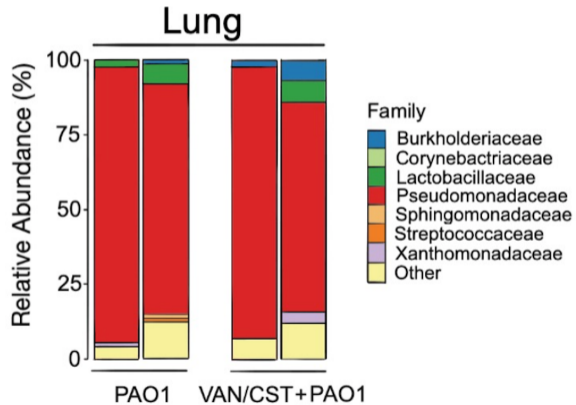**B**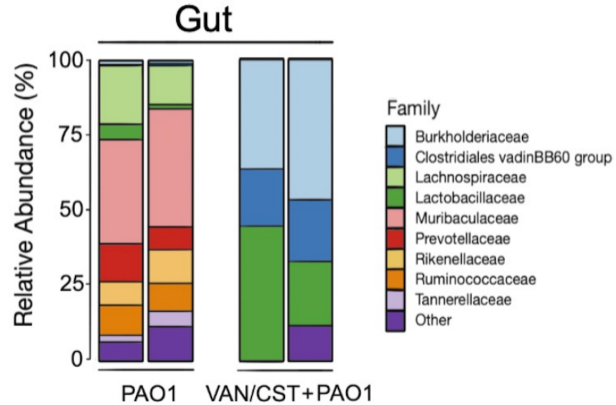

Supplement: Supplementary file 3 — Additional file 3 : Supplemental figure 3. Bacterial family as percentages of sequenced 16S rDNA in the lung and gut. Mice were treated or not by oral vancomycin (VAN), and colistin (CST) followed or not by fecal microbiota transplant (FMT) and infected or not by P. aeruginosa PAO1 (n = 2 per group). [file 13054_2020_3320_MOESM3_ESM.pdf]

**A**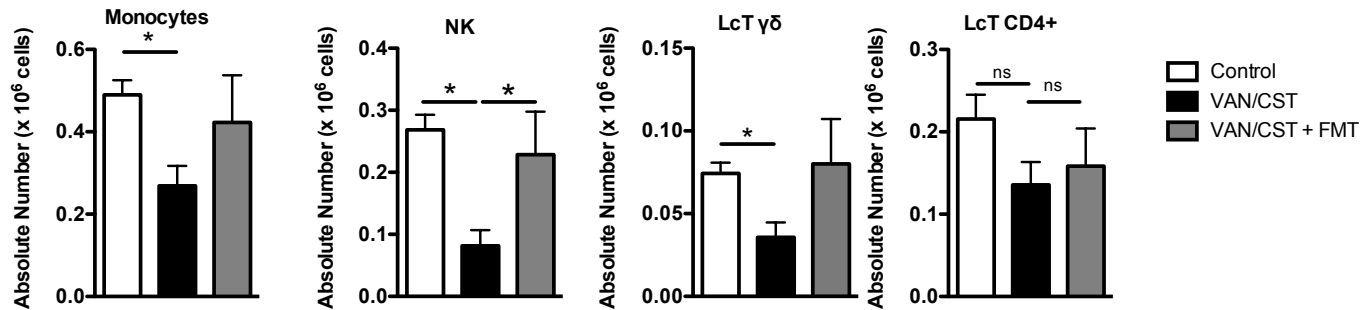**B**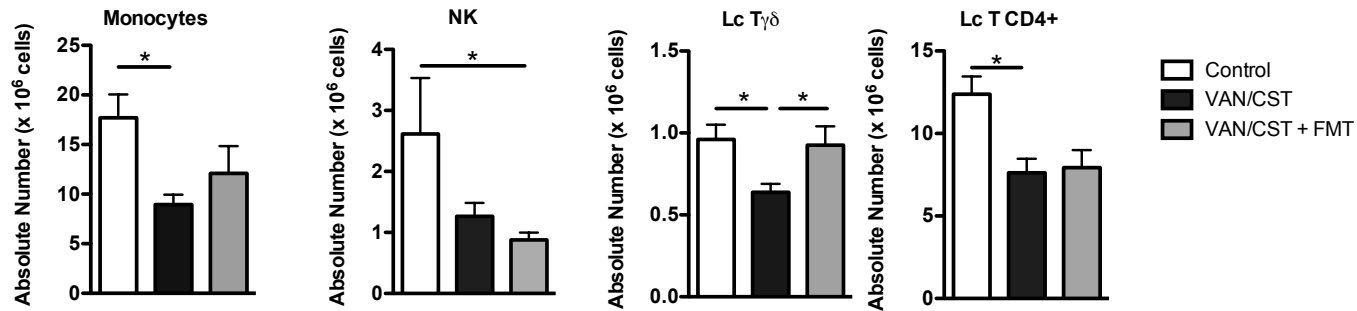

Supplement: Supplementary file 4 — Additional file 4 : Supplemental figure 4. Effects of oral non-absorbable antibiotics on lung and spleen immune cell populations. (A) Flow cytometry of immune cells from lung homogenates in mice treated or not (controls) with 7-days oral vancomycin/colistin (VAN/CST) followed by fecal microbiota transplant (VAN/CST + FMT) or not. (B) Flow cytometry of immune cells from spleen homogenates in mice treated or not (controls) with 7-days oral vancomycin/colistin (VAN/CST) followed by fecal microbiota transplant (VAN/CST + FMT) or not. All experiments, group size 5 mice per group, results are shown as mean ± SD; *: p < 0.05. [file 13054_2020_3320_MOESM4_ESM.pdf]

**A**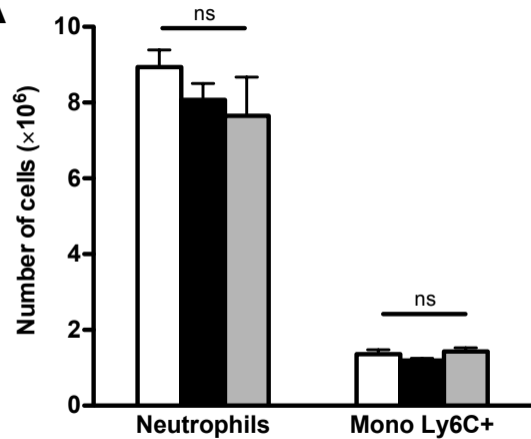**B**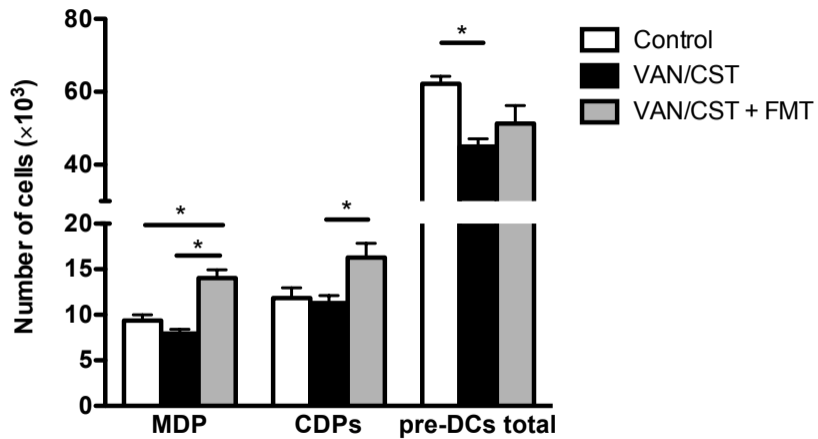

Supplement: Supplementary file 5 — Additional file 5 : Supplemental figure 5. Effects of oral non-absorbable antibiotics on hematopoietic factors and bone marrow monocyte/dendritic cell progenitors. (A) ELISA of hematopoietic factors in serum of mice treated or not (controls) with 7-days oral vancomycin/colistin (VAN/CST) followed by fecal microbiota transplant (VAN/CST + FMT) or not, (B) Flow cytometry of monocyte/DC progenitors in the bone marrow in mice treated or not (controls) with 7-days oral vancomycin/colistin (VAN/CST) followed by fecal microbiota transplant (VAN/CST + FMT) or not. For all experiments, group size 5 mice per group; results are shown as mean ± SD; *: p < 0.05. [file 13054_2020_3320_MOESM5_ESM.pdf]

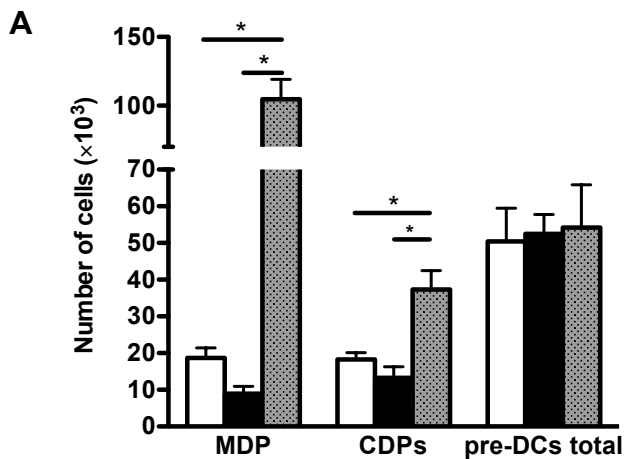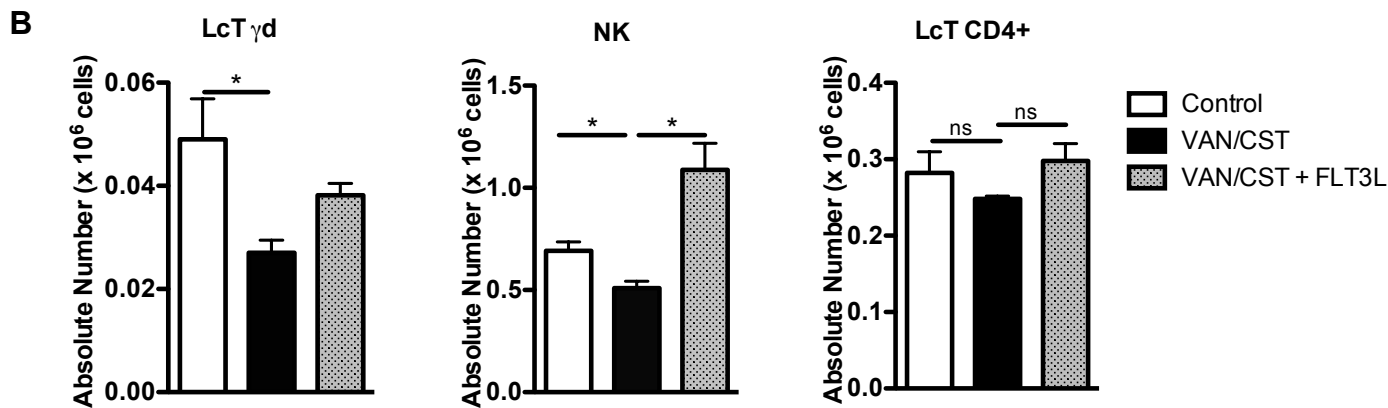

Supplement: Supplementary file 6 — Additional file 6 : Supplemental figure 6. Effects of Flt3-Ligand on lung immune cell populations and on outcomes of lung infection. (A) Flow cytometry of monocyte/DC progenitors in the bone marrow of mice treated or not (controls) with 7-days oral vancomycin/colistin (VAN/CST) followed by systemic Flt3-Ligand administration (VAN/CST + Flt3-L) or not, (B) Flow cytometry of immune cells from lungs of mice with 7-days oral vancomycin/colistin (VAN/CST) followed by systemic Flt3-Ligand administration (VAN/CST + Flt3-L) or not. For all experiments, group size 5 mice per group; results are shown as mean ± SD; *: p < 0.05. [file 13054_2020_3320_MOESM6_ESM.pdf]
