# Supplementary material for: Antibiotic-related gut dysbiosis induces lung immunodepression and worsens lung infection in mice
Source: Crit Care. 2020 Oct 15;24:611. doi: 10.1186/s13054-020-03320-8 (PMC7574210; doi:10.1186/s13054-020-03320-8)
Supplement: Supplementary file 7 — Additional file 7. [file 13054_2020_3320_MOESM7_ESM.docx]

**Supplementary material**

**Animals**

Specific pathogen-free C57BL/6 mice of 6-8 weeks old were purchased from Janvier Labs (Le Genest-Saint-Isle, France). Mice were housed in a controlled, specific pathogen-free environment. Mice were euthanized by lethal injection of 0.2mL of dolethal (Vétoquinol, Paris, France).

**Bacterial Strain**

*P. aeruginosa* PAO1 strain was grown overnight at 37°C in Luria-Bertani broth, with orbital shaking, harvested by centrifugation (3500rpm, 10min), and washed with sterile isotonic saline. Bacteria suspensions and inoculum standardization were then determined based on optical density at 600 nm and verified by serial dilutions and plating on Luria-Bertani agar (Fisher Scientific, Hampton, NH, USA).

**Bronchoalveolar lavage (BAL)**

Lungs from each experimental group were washed with a total of 1.5mL PBS. Recovered lavage fluid was centrifuged (1500rpm, 10min), the cellular pellet was washed twice with PBS. BAL samples were frozen at -80°C after collection for cytokine measurement. Cell counts were performed directly by optical microscopy.

**Bacterial burden**

Mouse lungs and spleens were homogenized in sterile containers with PBS. Lung and spleen homogenates were sequentially diluted and cultured on Luria-Bertani agar plates for 24 hours to assess bacterial load.

**Flow cytometry**

Antibodies and the permeation kits were purchased from BD. Cells harvested from BAL fluid and lungs were washed and incubated with appropriate dilutions of an antigen-presenting-cell antibody panel or a lymphoid-cell antibody panel for 30 min in PBS and then washed twice and resuspended in PBS 2% fetal calf serum. For each antibody, a control isotype was used for compensation. Cells were analyzed on a LSR Fortessa (BD Biosciences). Flow cytometry gating strategy to analyze AM, cDC1, cDC2, Inflammatory monocytes, monocytes, neutrophils, T lymphocytes (CD4^+^ and CD8^+^), NK cells, iNKT cells, NKT cells, and Tγδ cells, is illustrated in supplementary figure 2. Generated data were analyzed using FlowJo 8.7 (Tree Star, Stanford, CA).

**Bone marrow progenitor analysis**

Cell populations (CD45^+^ cells) were analyzed with a BD LSR Fortessa (BD Biosciences) according to the following cell surface phenotypes: (MDPs): Lin(*)^-^CD11b^-^CD117^+^CD135^+^CD115^+^; (CDPs) Lin(*)^-^CD11b^-^CD117^low^CD135^+^CD115^+^CD11c^-^; (pre-DCs): Lin(*)^-^CD11b^-^CD11c^+^MHCII^-^CD135^+^Sirpα^-/low^ and Siglec-H^-^Ly6C^-^ for cDC1-biaised pre-DCs or Siglec-H^-^Ly6C^+^ for cDC2-biaised pre-DCs; (cMoPs): Lin(*)^-^CD11b^-^CD117^+^CD135^-^CD115^+^Ly6C^+^ and (monocytes): Lin(*)^-^CD11b^+^CD115^+^Ly6C^high^ (inflammatory monocytes) or Ly6C^low/neg^ (patrolling monocytes). Dead cells were excluded by propidium iodide staining. For analysis, 5x10^5^-10^6^ CD45^+^ cells were acquired and the data were analyzed with FACSDiva or FlowJo software (TreeStar, US). Lin*: CD3, NK1.1, Ter119, CD45R/B220, Ly6G.

**Microbiome analysis**

Total DNA concentration was measured using Picogreen (Invitrogen). Global 16S gene DNA copy numbers were determined by a qPCR adapted from Maeda *et al*. (Maeda H et al. FEMS Immunol Med Microbiol. 2003 Oct 24;39(1):81-6), allowing for inhibition effect estimation and DNA concentration adjustment. The sequence region of the 16S rRNA gene spanning variable region V3–V4 was amplified using the broad-range forward primer: Bact-0341, 5′-CCT ACG GGN GGC WGC AG-3′ and reverse primer: Bact-0785, 5′-GAC TAC HVGGG TAT CTA ATC C-3′ (Klindworth A et al. Nucleic Acids Res. 2013;41(1):e1). Individual samples were barcoded, pooled to construct the sequencing library, and then sequenced using an Illumina Miseq (Illumina, San Diego, CA) generating paired-end 2 × 300 bp reads.

Microbiome data were analyzed using QIIME 2 2020.2 (Bolyen E et al. Nat. Biotechnol. 2019;37:852–7). Raw sequence data were demultiplexed and quality filtered with the q2-demux plugin followed by denoising with DADA2 (Callahan BJ et al. Nat. Methods. 2016;13:581–3). Taxonomy was assigned to amplicon sequence variants (ASVs) using the q2-feature-classifier plugin against the SILVA 132 99% database. Phylogenetic data clustered into ASVs were further analyzed using the phyloseq package. Contaminant DNA sequences were identified and removed using the decontam package (Davis NM et al. Microbiome. 2018;6:226). Both packages were used in R software version 3.6.3 (R Foundation for Statistical Computing, Vienna, Austria).
